# Supplementary material for: Si0.97Ge0.03 microelectronic thermoelectric generators with high power and voltage densities
Source: Nat Commun. 2020 Aug 31;11:4362. doi: 10.1038/s41467-020-18122-3 (PMC7458905; doi:10.1038/s41467-020-18122-3)
Supplement: Supplementary file 1 — Supplementary Information [file 41467_2020_18122_MOESM1_ESM.pdf]

Supplementary Information

for

$Si_{0.97}Ge_{0.03}$ microelectronic thermoelectric generators with high power and voltage densities

by Dhawan, *et al*.

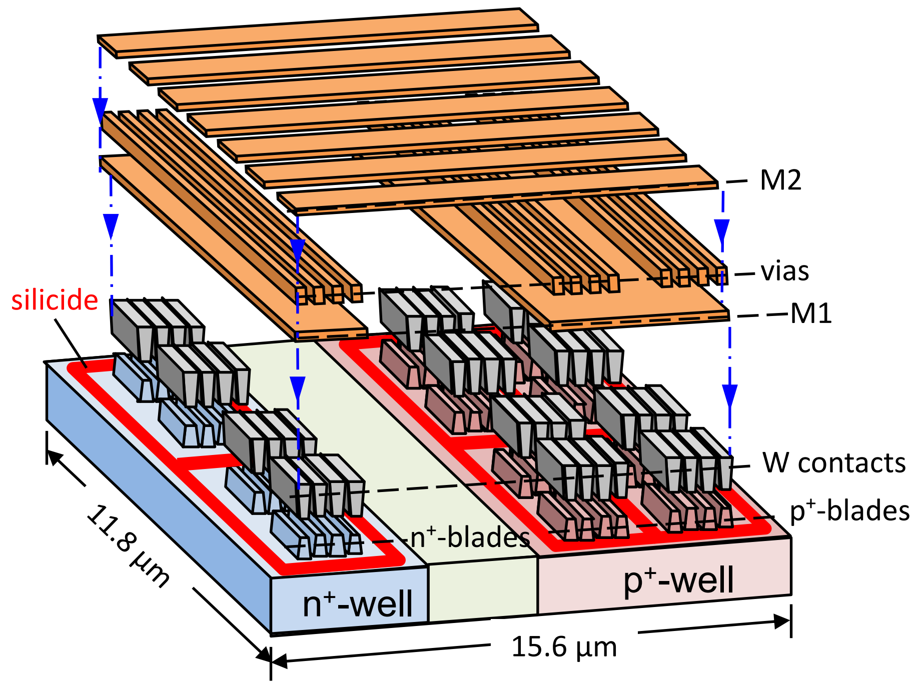

Fig. 1 | Expanded illustration (not to scale) of a thermocouple unit cell of the harvest mode µTEG used to generate the data shown in Figs. 3 and 4 of this paper. The doped wells, blades, tungsten (W) electrical contact, and copper bridging layers (labeled M1, vias, and M2) are depicted here, but not the heat exchanger layers as sketched in Fig. 2a of this paper. The layers have been offset vertically for clarity. The complete harvest mode µTEG thermopile consists of 640 such unit cells connected electrically in series over a 0.64 × 0.32 mm area.

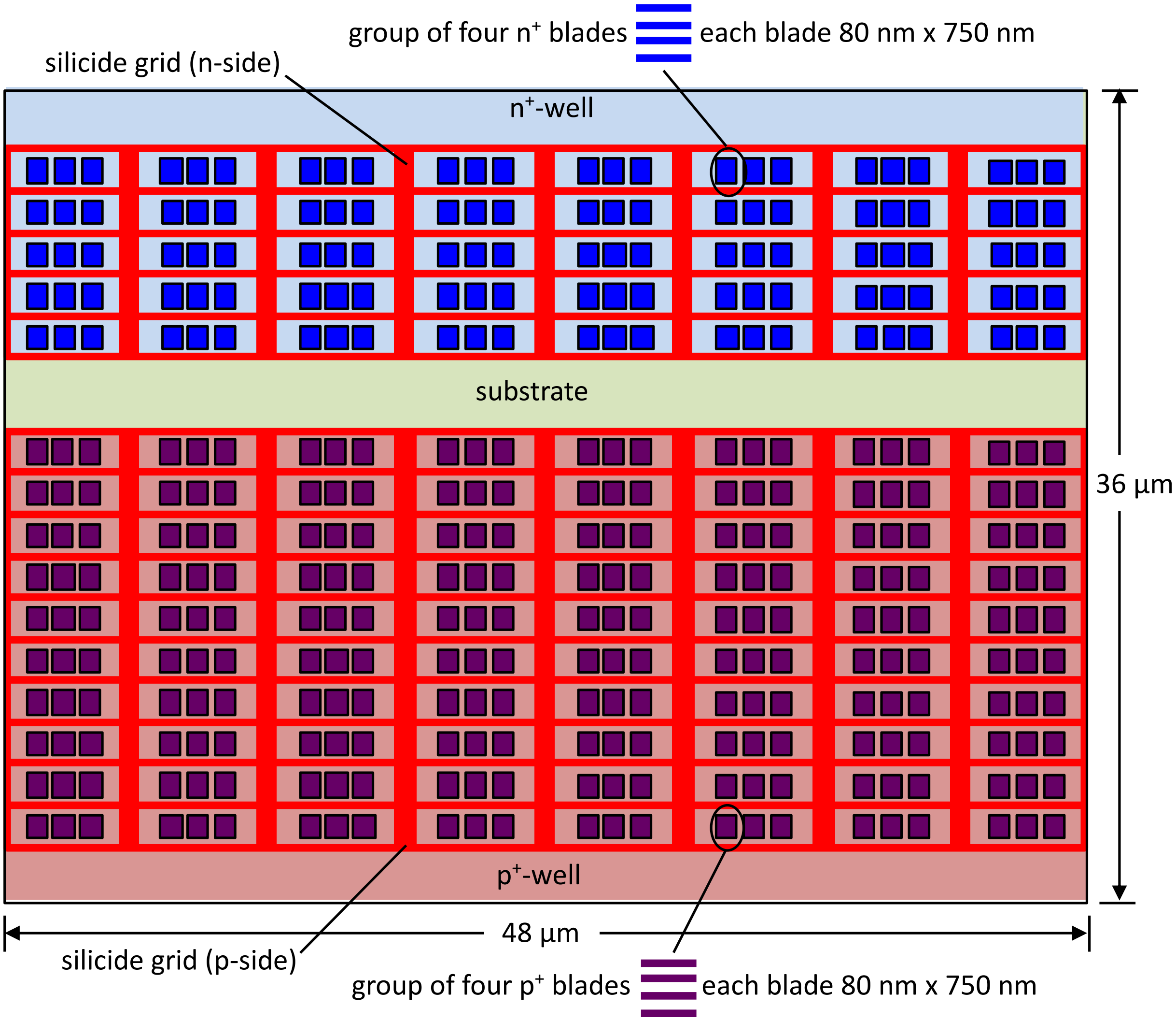

Fig. 2 | Plan view design drawing (to scale) of the thermopile layout of the test mode µTEG used to generate the data shown in Fig. 1 of this paper. The basic thermoelectric element is a blade of doped $Si_{0.97}Ge_{0.03}$ nominally 80 nm wide × 750 nm long × 350 nm tall. In this design, each cell in the silicide grid contains three repetitions of a four-blade group. The silicide grids serve as electrical contacts to the n^+ and p^+ wells from which the blade elements are etched. The heater serving as the hot thermal reservoir would be above the page and the substrate behind the page, so heat flows perpendicularly into the plane of the page.

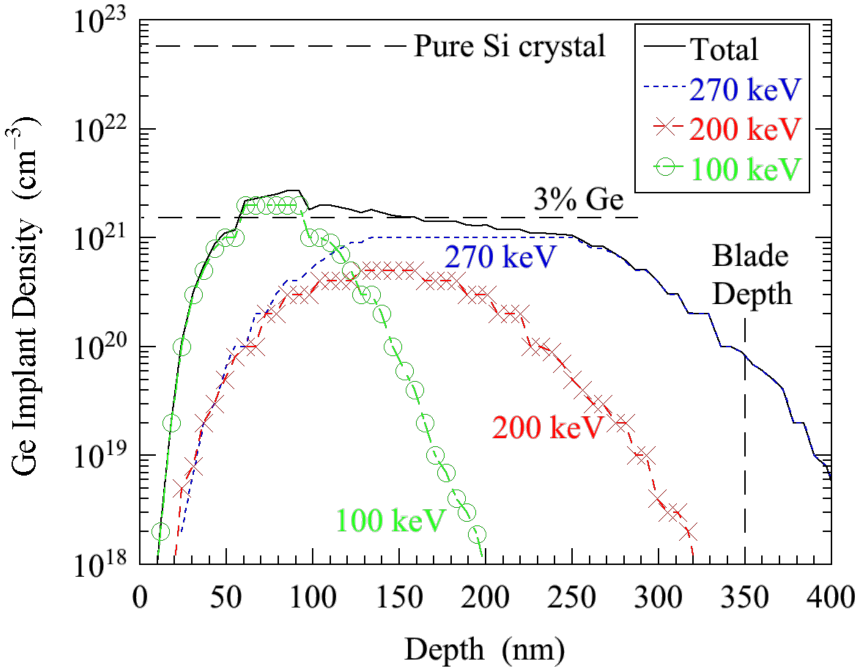

Fig. 3 | Simulation of Ge ion implant density as a function of depth into the Si wafer surface using the Transport of Ions in Matter (TRIM) simulator and the implant conditions given in Methods for the $Si_{1-x}Ge_x$ wafer with nominal $x = 0.03$. Shown are the simulated Ge density distributions for 100 keV (green dot-dash line with open circles), 200 keV (red dashed line with × symbols), and 270 keV (blue short dashed line) at the doses stated in Methods, and the total density (black solid line) equal to the sum of the densities from the three implant energies. Two horizontal black long-dashed lines indicate the atom number densities for pure crystalline Si (5×10^{22} cm^{-3}) and for 3% Ge in Si (1.5×10^{21} cm^{-3}). The vertical black long-dashed line indicates the nominal depth to which a thermoelectric blade element is etched into the wafer.
